# Radiochemical Approaches to Imaging Bacterial Infections: Intracellular versus Extracellular Targets

**DOI:** 10.3390/ijms20225808

**Published:** 2019-11-19

**Authors:** Justin D. Northrup, Robert H. Mach, Mark A. Sellmyer

**Affiliations:** 1 Department of Radiology, University of Pennsylvania, Philadelphia, PA 19104, USA; justin.northrup@pennmedicine.upenn.edu (J.D.N.); rmach@pennmedicine.upenn.edu (R.H.M.); 2 Department of Biochemistry and Biophysics, University of Pennsylvania, Philadelphia, PA 19104, USA; 3 Institute for Translational Medicine and Therapeutics, University of Pennsylvania, Philadelphia, PA 19104, USA

**Keywords:** infection imaging, molecular imaging, positron emission tomography, antibiotics, radiochemistry, chemistry

## Abstract

The discovery of penicillin began the age of antibiotics, which was a turning point in human healthcare. However, to this day, microbial infections are still a concern throughout the world, and the rise of multidrug-resistant organisms is an increasing challenge. To combat this threat, diagnostic imaging tools could be used to verify the causative organism and curb inappropriate use of antimicrobial drugs. Nuclear imaging offers the sensitivity needed to detect small numbers of bacteria in situ. Among nuclear imaging tools, radiolabeled antibiotics traditionally have lacked the sensitivity or specificity necessary to diagnose bacterial infections accurately. One reason for the lack of success is that the antibiotics were often chelated to a radiometal. This was done without addressing the ramifications of how the radiolabeling would impact probe entry to the bacterial cell, or the mechanism of binding to an intracellular target. In this review, we approach bacterial infection imaging through the lens of bacterial specific molecular targets, their intracellular or extracellular location, and discuss radiochemistry strategies to guide future probe development.

## 1. Introduction

Bacterial infections are a major biomedical problem, and at least six million global deaths were due to infectious diseases in 2017 [1]. There are a variety of types of infections, from endemic, community-acquired infections typical of the developing world, to rare atypical infections in patients with complex medical problems seen in modern tertiary care centers. In the latter case, many antimicrobial-resistant infections are attributable to hospital-acquired organisms (nosocomial infections), resulting in prolonged in-patient stays and increased costs to both the individual and society at large [2]. The increased individual cost associated with an antibiotic-resistant infection may vary from several hundred dollars to over a hundred thousand dollars depending on the type of bacterium and severity of the infection. More broadly, the total cost-burden on the gross domestic product is estimated to be in the tens of billions of dollars in lost productivity [2]. The therapeutic and prophylactic use of antimicrobials in humans has undoubtedly spurred some of this resistance, in addition to antimicrobial use in biomedical research, as well as other anthropogenic activities such as farming and agriculture [3]. Moreover, the overuse of antibiotics in human patients has been highlighted by the development of highly resistant strains of lethal bacteria, for example, *Clostridium difficile* [3]. While precision human imaging tools have been rapidly developed for cancer diagnostics, the ability to specifically image bacterial infection in people has lagged behind. Such molecular tools would allow better management of antibiotics in human patients, especially in the hospital setting where empiric use is common, and would allow tailored therapies.

Given that bacteria come from a different phylogenetic kingdom than humans, the development of bacterial targeted imaging agents should be a relatively straightforward process. However, over the last 50 years, an infection specific agent has proven elusive [4,5]. If more probes were developed to target specific strains or classes of bacteria, this could, in turn, lead to a more rapid and specific treatment, resulting in improved clearance of the infection. Furthermore, the use of a narrower treatment could, in turn, curb the rise of multidrug-resistant organisms. In order to appropriately treat a patient’s infection, a physician may first determine what the causative organism is. The current microbiological methods of diagnosing bacterial infections include microscopy, culture techniques, nucleic acid amplification (PCR), and mass spectrometry, usually after tissue sampling or biopsy [6]. There is a multitude of issues with these methods: Ex vivo cultivation of the bacterium in question (anaerobes); sample contamination and error; single location of tissue sampling, and use of a single time point that may not indicate temporal changes inherent in the highly dynamic infection pathobiology. Additionally, imaging techniques are often applied in conjunction with microbiological methods. Current imaging methods of diagnosing bacterial infections often include magnetic resonance imaging (MRI), ultrasound (US), computed tomography (CT), positron emission tomography (PET), and single-photon emission computed tomography (SPECT). Many of these methods rely on simple vascular flow (contrast enhancement) and are unable to differentiate bacterial infection from sterile inflammation, anatomic tissue changes, due to cancer or cancer-related treatments (e.g., radiation), or even other infections (e.g., viral or parasitic) [7].

With these limits in mind, and given the sensitivity afforded by nuclear imaging, several research groups have been developing new radiotracers, purported to be capable of determining whether a particular site of uptake is due to cancer or a microbial infection. These investigational radiotracers target infections through a variety of approaches, including labeled antibiotics, antimicrobial peptides, and metabolic substrates [6,8,9,10,11]. Some of the past radiotracers have not worked, despite some initially promising results [4,12]. While a new generation of tracers is under review or moving into clinical trials [12,13,14], next-generation approaches should consider the structural features of the protein-binding site, and more macroscopically, the subcellular locale of the target (i.e., whether the target is intracellular or extracellular). The rational design of these new tracers must also consider the size and structure of the probe itself, as well as its pharmacokinetics and biological half-life (t_½_) in conjunction with the radiochemical t_½_. Many imaging agents developed from antibiotics have been chelated to technetium-99m, in part, due to the ease of labeling, using off-the-shelf kits. However, achieving bacterial specificity with these probes has been challenging, with the site of labeling in some cases potentially interfering with proper binding to the target [15,16]. This review discusses an approach that stresses the location of the target, and then retro-synthesizes the best radiochemical approach based on the size of the probe. For instance, if an extracellular domain is to be targeted, a radionuclide that requires chelation ought to be considered given the ease of generator derived radioisotopes, such as gallium-68 or technetium-99m. Whereas, covalent modifications are more difficult to achieve radiochemically, their ability to penetrate bacterial cells when paired with a small molecule probe opens the spectrum of intracellular targets [12,13,17]. It, therefore, seems that location of the target should be an important factor to consider when developing new probes, along with other radiochemical factors, such as the ease of labeling, the half-life of the radioisotope, and modality of imaging (PET or SPECT).

## 2. Radionuclide Choice: Chelation vs. Covalent Attachment

There are a wide variety of radionuclides that can be used for radiolabeling a compound, but they fall into two main categories: Metals which must be chelated by some part of the molecule, and nonmetals which are covalently attached. Shown in Table 1 are some of the most used radionuclides for imaging purposes. This includes the common positron-emitting nuclides carbon-11, fluorine-18 and gallium-68, as well as the γ-emitting nuclides technetium-99m and iodine-123. Discussed in this section is the generation of some clinical radionuclides, their common methods of inclusion into radiopharmaceuticals, and a common clinical application for these radionuclides. This section is not meant to be an exhaustive review of radiochemistry or radiopharmaceutical chemistry (for an exhaustive review, see Radiopharmaceutical Chemistry [18]). Rather, the goal is to highlight standard approaches and considerations for developing bacterially-targeted radiochemical probes.

### 2.1. Chelation of Radionuclide

Radioisotopes available for either diagnostics or therapeutics are often metals which include copper-64, zirconium-89, gallium-68, yttrium-90, and technetium-99m. These metals cannot be attached covalently to a molecule; instead, they require some form of chelation for inclusion in the molecule. This could be in the form of a macrocyclic chelator attached to the pharmacophore by a linker, or direct chelation by the molecule using the pharmacophore atoms. The latter poses a concern, since the pharmacophore atoms used for chelation usually play an integral role in the in vivo targeting properties. Often times these chelates change the pharmacodynamics and/or pharmacokinetics of the molecule in question, which should be considered when developing the radiopharmaceutical.

**Figure 1:**
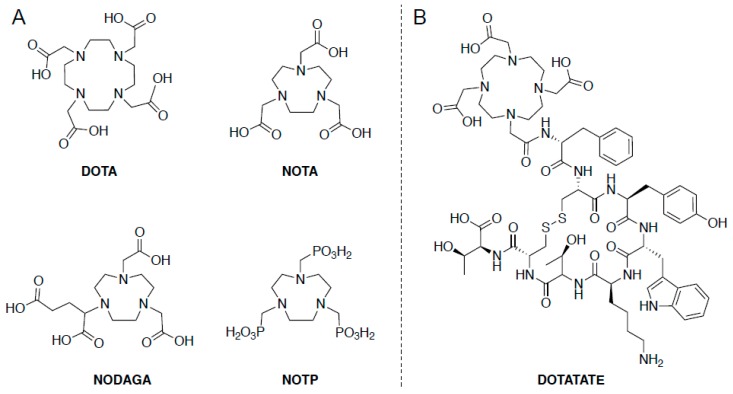
(**A**) Common moieties used for chelation; (**B**) Chemical structure for DOTATATE.

A radionuclide that has recently gained more widespread use is gallium-68 which could be useful for infection PET imaging. The reason for the interest is two-fold. First, gallium-68 is a positron-emitting radionuclide, thus, allowing for its use in PET applications, and second is the ease of gallium-68 generation from the decay of germanium-68 [21]. Germanium-68 has a T_½_ of 271 days, which makes it a useful parent isotope for a generator, as this longer T_½_ means a generator need only be purchased once a year. The increase in the use of gallium-68 and other radiometals has led to the use or development of new macrocyclic chelators, including DOTA, NOTA, NODAGA, and NOTP (Figure 1A). As an example of the growing use of chelators for PET imaging, one of the most well-known gallium-68 radiopharmaceuticals is [^68^Ga]Ga-DOTA-Octreotide, or DOTATATE (Figure 1B). DOTATATE targets the somatostatin receptors (specifically SSR2), which are overexpressed in numerous malignancies, including breast, lung, lymphatic, and neuroendocrine tumors [22]. Since these receptors are transmembrane proteins with extracellular domains, the chelated DOTATATE is still able to bind to its target of interest, an important aspect of any chelated radiotracer.

One of the most widely utilized radionuclides worldwide is technetium-99m, a γ-emitting nuclide which is used in approximately 25 million yearly procedures [23]. Technetium-99m has a T_½_ of 6 h, and is the daughter radionuclide of molybdenum-99. Typically, technetium-99m is obtained from a ^99^Mo/^99m^Tc generator, aiding its worldwide application. The molybdenum is adsorbed onto an aluminum oxide column in the form of [^99^Mo]MoO_4_^2−^ (^99^Mo-Molybdate), which then decays into [^99m^Tc]TcO_4_^−^ and can be eluted from the generator using a saline solution. The [^99m^Tc]TcO_4_^−^ must then be reduced and combined with a suitable ligand. A crucial note is that technetium has oxidation states ranging from −1 to +7, and ligand complexes with Tc have been found as tetrahedral (4 coordinate), tetragonal pyramidal (5 coordinate), octahedral (6 coordinate), capped octahedral (7 coordinate), and pentagonal bipyramidal (8 coordinate). All of these different structural possibilities need to be considered when developing ^99m^Tc-based radiopharmaceuticals, as the differences in structure can cause drastic changes in physical and biochemical properties.

### 2.2. Covalent Attachment of Radionuclide

While many metals are available for chelation, there are relatively fewer radionuclides available for covalent attachment to a diagnostic or therapeutic probe, with the majority being halides. This includes carbon-11, fluorine-18, bromine-77, and the various isotopes of iodine (I-123, I-124, I-125, I-131). Whereas, chelated radionuclides typically possess similar moieties regardless of the metal to be chelated, covalent attachment of radionuclides usually requires the generation of a variety of precursors, depending on the radionuclide to be attached. In the case of carbon-11, which is typically added as the electrophilic [^11^C]methyl iodide, the precursor must be sufficiently nucleophilic [24]; whereas, in the case of [^18^F], the precursor is usually an electrophile to allow for the addition of the nucleophilic [^18^F]fluoride anions [25]. In the case of the other halogens, Br or I, this typically proceeds via nucleophilic aromatic substitution of tributyltin (SnBu_3_) derivative [21,26].

**Figure 2:**
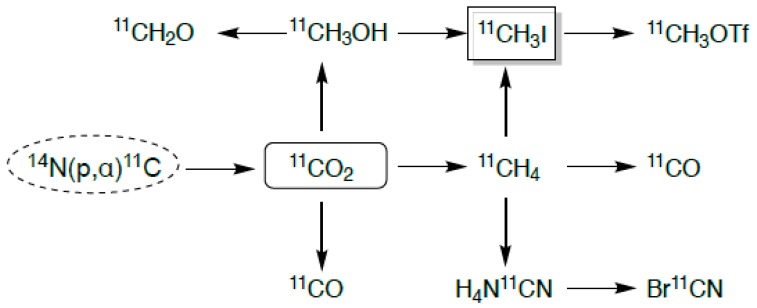
Production routes for common carbon-11 labeling derivatives.

Carbon-11 is a positron-emitting radionuclide with one of the shorter t_1/2_ of ~20 min, and is produced by proton bombardment of nitrogen gas in the nuclear reaction ^14^N(p,α)^11^C [19]. It is generally produced as either carbon dioxide ([^11^C]CO_2_) or methane ([^11^C]CH_4_) [24,27], depending on the primary precursor desired; however, the vast majority of carbon-11 is generated as carbon dioxide, a useful starting point for many labeling methods (Figure 2**)**. The [^11^C]CO_2_ can be reduced to [^11^C]methane, and the unreacted [^11^C]CO_2_ is separated from the [^11^C]methane by a carbon dioxide trap. The [^11^C]methane can then be transformed into a reactive species, usually methyl iodide ([^11^C]CH_3_I) or cyanide ([^11^C]HCN). Methyl iodide is a useful material for electrophilic addition, as it is reactive to even weak nucleophiles. Due to its relatively short T_½_, carbon-11 derivatives are not commonplace in the clinic. Some uses for carbon-11 is its addition to amino acids, such as methionine, or derivatives of amino acids, such as choline. l-[*Methyl*-^11^C]methionine is typically used for imaging of brain tumors, where the use of [^18^F]fluorodeoxyglucose ([^18^F]FDG, see below) is hindered by high background accumulation in the brain [27]. Another use is in [^11^C]acetate, which has shown use in myocardial perfusion and oxygen metabolism, and more recently, in oncology for prostate cancer, renal cell carcinoma, and brain tumors [28].

**Figure 3:**
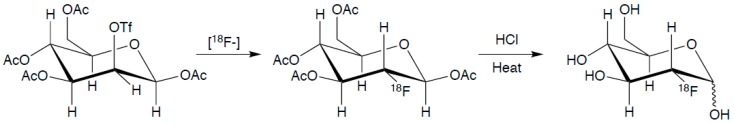
Chemical structures of [^18^F]FDG and its triflate precursor.

Fluorine-18 is the most widely used positron-emitting radionuclide for imaging [25], usually in the form of [^18^F]fluorodeoxyglucose ([^18^F]FDG). Fluorine-18 has a t_1/2_ of 110 min, making it much more useful from a clinical imaging perspective than carbon-11 because it can be shipped over short distances allowing wider access and improved biodistribution/pharmacokinetic characteristics. Fluorine-18 is produced by the proton bombardment of heavy water ([^18^O]H_2_O) in the following nuclear reaction: ^18^O(p,n)^18^F [19], which means an oxygen-18 nucleus captures a proton and ejects a neutron. The reaction is not very efficient, with a large percentage of [^18^O]H_2_O remaining. The solution containing the [^18^F]fluoride anions is transferred through an ion exchange cartridge, which captures the ions, and allows recovery of unreacted [^18^O]H_2_O. After recovery of the [^18^O]H_2_O, the [^18^F]fluoride anions are eluted from the ion exchange cartridge with a counterion, usually carbonate (CO_3_^2−^). For the production of FDG, the fluoride ion needs to be anhydrous, as water can compete with the [^18^F]fluoride anion when displacing the triflate (OTf, Figure 3). [^18^F]FDG is a metabolic tracer used for many different clinical applications, including diagnosis and monitoring of sarcoidosis [29], and most commonly for the detection of cancers [30,31]. Since cancer cells are continually dividing, they require more nutrients than normal tissue. This means that a cancerous lesion will accumulate more [^18^F]FDG than the surrounding tissue, allowing for high-contrast PET/CT imaging of the lesions. Unfortunately, one of the main drawbacks of [^18^F]FDG is the fact that it is nonspecific, and will show increased accumulation in any area with increased metabolic activity, including infection and sterile inflammation [32].

Now that we have briefly reviewed the radiochemical approaches available, we will focus next on optimal strategies for the next generation of radiochemical probes for infection. As we highlight these various methods, it is important to keep in mind the possibilities for radionuclide inclusion (chelation or covalent assemblies), whether these probes are targeting intracellular or extracellular molecules, and the T_½_ of the radionuclide vs. clearance of the radiotracer.

## 3. Intracellular Targets

Many antibiotic targets are located within the bacterial cell, which means the radiopharmaceutical must be able to cross the bacterial cell wall and/or plasma membrane in order to identify a site of infection. In this next section, we will discuss the several classes of intracellularly active molecules, including β-lactams, macrolides, fluoroquinolones, and anti-folates, followed by a brief discussion of metabolic tracers (reviewed elsewhere [14].)

### 3.1. Inhibitors of Peptidoglycan Cross-Linking–β-lactams

Β-lactam antibiotics are still one of the most widely used. From the original penicillin, through modern cephalosporins, these broad-spectrum antibiotics are crucial drugs for combating infection. These drugs inhibit the family of proteins known as penicillin-binding proteins (PBP). PBP cross-link the peptidoglycan layer of the bacterial cell wall, and inhibition of this process leads to cell death. Many research groups have labeled these broad-spectrum antibiotics for use as imaging agents, albeit with limited success. This lack of success as imaging agents could stem, in part, from the use of ^99m^Tc-chelation, as opposed to covalent attachment of a radionuclide [8,9]. Another possible pitfall of using β-lactams as imaging agents is the prevalence of microbial-resistance in the form of β-lactamases though horizontal gene transfer for example [33].

**Figure 4:**
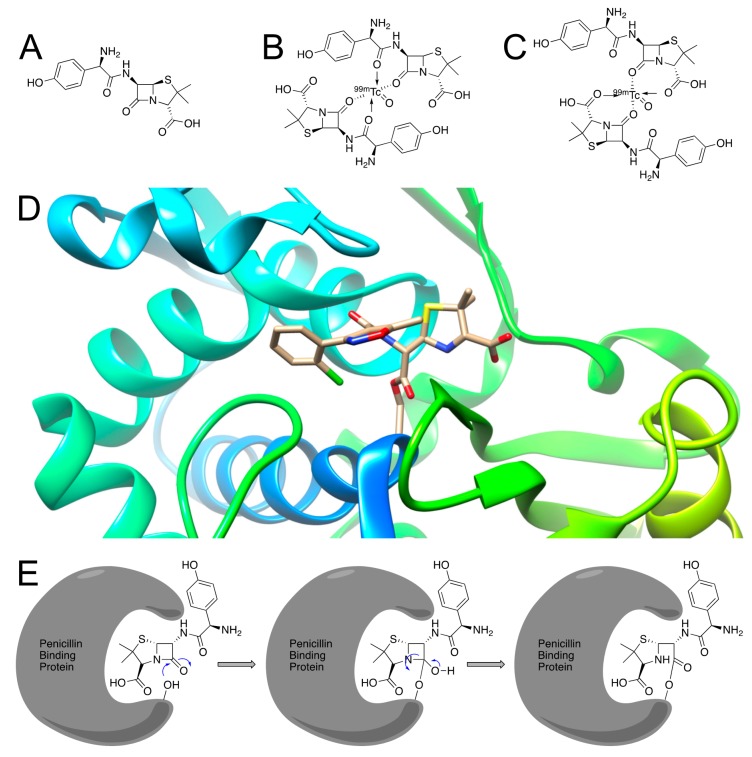
(**A**) Amoxicillin; (**B**) Proposed structure for ^99m^Tc-labeled amoxicillin; (**C**) Another potential structure for ^99m^Tc-labeled amoxicillin; (**D**) Crystal structure of a similar β-lactam antibiotic (cloxacillin) bound to PBP 53 (PDB ID: 3MZD) [35]; (**E**) Mechanism for amoxicillin-binding to a target PBP [11].

Amoxicillin (Figure 4A) is one of the most widely prescribed β-lactam antibiotics, and as such, would seem to be an enticing choice for functionalization into a radiotracer. One such method involved its chelation to technetium-99m [15]. Javed et al. indicated a target/non-target (T/NT) ratio of 3.5 ± 0.08 at 1 h post injection and proposed a possible structure (Figure 4B), but there exists another possible structure (Figure 4C). However, both of these structures have the possibility of sterically hindering the binding of amoxicillin to PBPs [34,35] (Figure 4D,E).

There are several other β-lactam antibiotics which have been chelated to technetium-99m, including cefazolin, ceftizoxime, and ceftriaxone. ^99m^Tc-labeled ceftriaxone has been studied most extensively, but with conflicting data. In mouse models, some groups show T/NT of 4.5 and 5.67 ± 0.6 against *E. coli* [34] and *S. aureus* [35], respectively; whereas, others in mouse models against *S. aureus* have shown T/NT of 3.39 for infected muscle, with high uptake in both heat-killed bacteria and sterile inflammation (T/NT of 3.12 ± 0.35 and 2.48 ± 0.45, respectively.) [36] These molecules most likely chelate technetium-99m in a way analogous with amoxicillin. We speculate that the impaired binding is the main reason for these borderline results, and we are not aware of any research which performed minimum inhibitory concentration (MIC) assays with either the ^99m^Tc-labeled β-lactams or the nonradiolabeled Tc-chelated β-lactams. Such data would be interesting to verify if the antibiotic retains a similar MIC to the non-labeled antibiotic. The conflicting results of these radiolabeled antibiotics should give pause to anyone interested in using a ^99m^Tc-chelated β-lactam or any intracellularly targeted probes. However, it would be interesting to consider covalently attached radionuclides probe for PBPs if the binding affinities were high enough (nanomolar range).

### 3.2. Inhibitors of Protein Synthesis–Macrolide Antibiotics

Macrolide antibiotics are natural products that typically consist of a 14-16-membered macrocyclic lactone ring, upon which one or more sugars are usually attached. Macrolides are effective primarily against Gram-positive bacteria, and inhibit protein synthesis by binding to the large subunit of the bacterial ribosome. While incredibly effective antibiotics, few macrolides have been used as radiotracers, most likely due to their large size and the difficulty of chemical modification.

**Figure 5:**
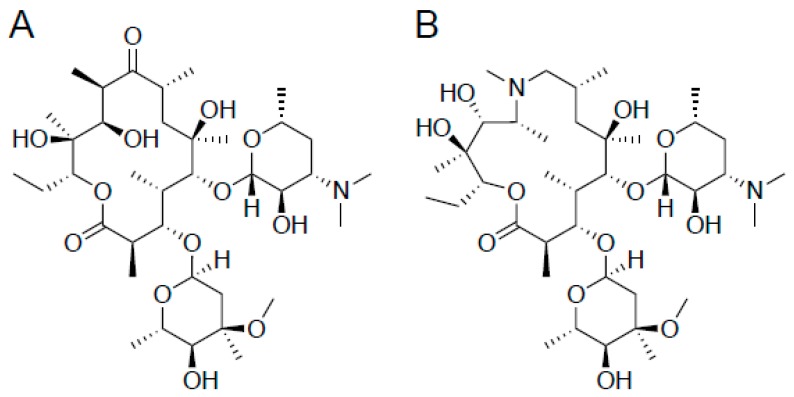
Chemical structures of two common macrolide antibiotics, erythromycin (**A**) and azithromycin (**B**).

Two common macrolides are erythromycin and azithromycin (Figure 5). One attempt to label a macrolide was through the use of erythromycin for chelation of technetium-99m, where the authors were able to achieve 97% labeling, and T/NT ratios of 5 ± 0.6. Unfortunately, biodistribution studies of ^99m^Tc-labeled erythromycin in albino mice were unable to differentiate septic from aseptic inflammation [37].

Other macrolide antibiotics that were labeled with technetium-99m include vibramycin, azithromycin, and clarithromycin. ^99m^Tc-labeled vibramycin was tested against *S. aureus* in a rat model, and showed T/NT ratios of 2.64, 2.15, and 1.80 against live bacteria, heat-killed bacteria, and sterile inflammation, respectively. It, therefore, was not further investigated as an imaging agent. ^99m^Tc-labeled azithromycin and ^99m^Tc-labeled clarithromycin, on the other hand, showed higher accumulation in infectious muscles as compared to controls. ^99m^Tc-labeled azithromycin was tested against *S. aureus* in a mouse model, achieving a max T/NT ratio of 6.20 ± 0.12 in live bacteria at 2 h. At the same time points against heat-killed bacteria and sterile inflammation, T/NT ratios were 3.16 ± 0.14 and 2.60 ± 0.12, respectively [38]. Similarly, ^99m^Tc-labeled clarithromycin was tested against *S. aureus* in a mouse model, showing T/NT ratios of 7.33 ± 0.13, whereas, against heat-killed bacteria and sterile inflammation, the T/NT were 3.1 ± 0.13 and 3.26 ± 0.12, respectively [39]. For both azithromycin and clarithromycin, the higher uptake in live cells is a promising result; however, the high signals in both heat-killed and sterile inflammation are a concern.

Similar to the β-lactam antibiotics, the use of the antibiotic as a structural basis for chelation provides limited success. While inherently difficult, modification of the peripheral sugars for radioflourination might yield new potential radiotracers; however, the inherent difficulty in macrolide synthesis might preclude their use as radiotracers with covalently bound radionuclides.

### 3.3. Inhibitors of DNA Synthesis–Fluoroquinolones

Fluoroquinolones are effective broad-spectrum antibiotics based on the 4-quinolone core structure. These antibiotics bind to either DNA topoisomerases or gyrases, preventing the unwinding of DNA leading to cell death. They are effective antibiotics against both Gram-positive and Gram-negative bacteria. A concern with the fluoroquinolone class of antibiotics is the propensity for adverse side-effects. In 2008, the US FDA added a black box warning for the increased risk of tendon damage, and in 2016, this was amended to include serious side effects involving muscles, joints, nerves, and the central nervous system. Given that tracer quantities are much lower than typical therapeutic doses, these side-effects should not pose an issue.

The first radiolabeled antibiotic tested in humans was ^99m^Tc-labeled ciprofloxacin, commonly known by the trade name “Infecton”. In a rat model, it showed high sensitivity, but low specificity in targeting experiments, while having superior biodistribution with renal clearance [40,41,42,43,44]. Ciprofloxacin was further conjugated to gallium-68 via a propylamine moiety, and was able to image *S. aureus* [45]. Unfortunately, testing of ^99m^Tc-labeled ciprofloxacin for infection localization in higher-order species, such as rabbits, dogs, camelids, or swine resulted in controversial data [46,47,48,49]. Furthermore, [^18^F]ciprofloxacin was synthesized as an alternative to ^99m^Tc-labeled ciprofloxacin; however, it was shown to not be specific for imaging infections, casting some doubt on radiolabeled antibiotics in general [50,51].

Second and third generation fluoroquinolones have also been labeled with technetium-99m and fluorine-18. Enrofloxacin was labeled with technetium-99m, but was unable to distinguish between infected muscle and control inflammation in a rat model [52,53]. Similarly, ^99m^Tc-Norfloxacin tested in rats showed modest T/NT ratios of ~3.0 and 1.0 for infected tissue and controls, respectively [54,55]. Fleroxacin is a third-generation fluoroquinolone which was labeled with fluorine-18, and evaluated in E. coli infected mice, rats, and rabbits [56]. Unfortunately, T/NT ratios were low, and [^18^F]fleroxacin was deemed a poor PET imaging agent. The fluoroquinolone antibiotic imaging story has led the infection imaging field away from antibiotics; however, we suggest that if the binding mechanisms are well described, and covalent radiochemistries are used, radiolabeled antibiotics may yet have some promise.

### 3.4. Inhibitors of Folic Acid Synthesis–Sulfonamides and Trimethoprim

**Figure 6:**
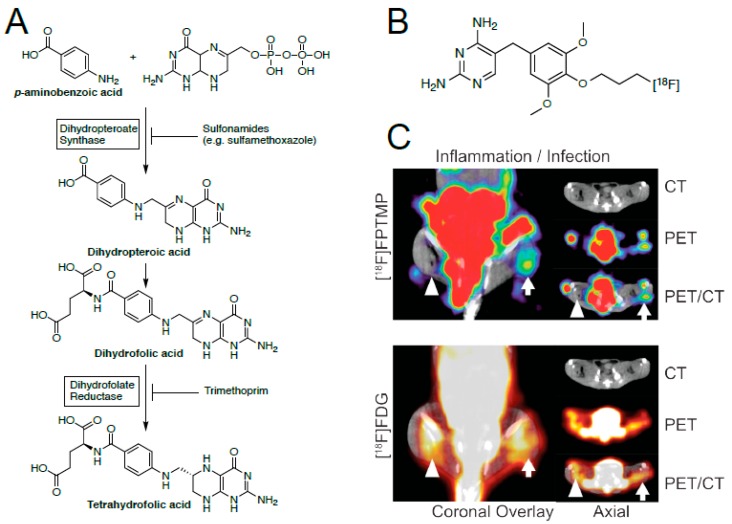
(**A**) Enzymatic pathway for the production of tetrahydrofolic acid from *p*-aminobenzoic acid. (**B**) Chemical structure of [^18^F]FPTMP. (**C**) A representative animal after [^18^F]FPTMP, ~200 μCi i.v., shows uptake in the infected hindlimb muscle (arrow) 4 h after infection, but not in the area of turpentine injection (arrowhead). Next-day imaging with FDG, ~300 μCi i.v., shows uptake in both infection and chemical inflammation 1 h after injection (adapted from Sellmyer, et al., 2017).

Folic acid (tetrahydrofolate) is an essential nutrient for nucleic acid synthesis in both prokaryotic and eukaryotic organisms. Mammals possess an active transport system for folic acid, which is capable of transporting it across the membrane; whereas, in most microorganisms, folic acid is synthesized *de novo* from *para*-aminobenzoic acid (PABA) and dihydropterin pyrophosphate [57]. A common antibiotic cocktail used to inhibit this synthesis in microorganisms is Bactrim, a formulation of sulfamethoxazole (SMX) and trimethoprim (TMP). SMX inhibits the enzyme dihydropteroate synthase, whereas, TMP inhibits dihydrofolate reductase (DHFR, Figure 6). Importantly, trimethoprim has a 10^3^–10^4^ higher affinity for bacterial DHFR (eDHFR) than the human analogue. Thus, TMP could possibly allow high enough target to the background for high-contrast imaging of a live in vivo bacterial infection.

Our group recently developed two antibiotic-derived radiotracer based on TMP, which was modified to enable radiolabeling with either carbon-11 [58] or fluorine-18 [12]. Like most covalently derived radiotracers, [^18^F]fluoropropyl-trimethoprim, [^18^F]FPTMP and [^11^C]TMP require chemical synthesis of the precursors. Despite the effort that entails, the dimethoxy-phenol is our common GMP precursor for the carbon-11 and fluorine-18 radiotracer (via a two-step radiosynthetic approach). [^18^F]FPTMP showed a modest T/NT ratio of 2.7, with little to no increase in uptake for sterile inflammation. This is compared to FDG, which showed similar uptake in infection and inflammation (Figure 6B). More studies with [^11^C]TMP (currently in clinical evaluation) and [^18^F]-TMP are ongoing.

Subsequently, another group attempted chelation of trimethoprim, ^99m^Tc-labeled TMP [16]. Unfortunately, this version of trimethoprim requires chelation of the technetium-99m utilizing the 2,4-diaminopyrimidine, which is an integral aspect for trimethoprim-binding to DHFR. [59] The pyrimidine enters deep into the DHFR binding pocket, so any modification at this site will most likely reduce binding, much like a ^99m^Tc-chelated β-lactam. Indeed, ^99m^Tc-labeled TMP shows no statistically significant difference at 30 min or 240 min post injection. This again highlights the need to consider radioligand-binding and location of the target before considering new approaches.

### 3.5. Metabolic Tracers

While this review focuses on antimicrobial targeted-tracers, it is important to note the other types of targeted and non-targeted tracers. As mentioned above, [^18^F]FDG is a nonspecific metabolic tracer, which shows increased signal wherever there is increased metabolic activity, be it from cancer, inflammation, or infection. This makes it a useful tool for determining if there is indeed an underlying issue, and where it might be located; however, it cannot distinguish one type of disease from another. Other groups have focused on metabolic tracers that are bacterially specific. These include [^11^C]*para*-aminobenzoic acid (PABA) [60] and a derivative 2-[^18^F]-*para*-aminobenzoic acid [61], used for the biosynthesis of tetrahydrofolate (Figure 6); 2-deoxy-2-[^18^F]fluoro-D-sorbitol [14,17], a common energy source for bacteria; and 6″-[^18^F]fluoromaltotriose [13,62], another energy source specific to bacteria. These three tracers have shown T/NT ratios of 7.9, 7.9 and 3.4, respectively; thus, all three of these tracers have potential as infection specific imaging agents, and these agents are currently moving toward clinical translation with early promising results [14,63].

## 4. Extracellular Targets

In the previous section, we focused on intracellularly targeted antibiotics, with multiple examples of chelated radiotracers that may affect target binding (e.g., ^99m^Tc-labeled amoxicillin and ^99m^Tc-labeled trimethoprim [15,16]). While most antibiotics have intracellular targets, other bacterial specific molecules exist that allow for extracellular targeting. Whereas, it might be difficult for chelated molecules to enter a bacterial cell, the use of these derivatives for chelation of radionuclides would be well suited for extracellular targeting. This includes the use of both antibodies and antimicrobial peptides, and in keeping with the antimicrobial theme of this review, we will focus mainly on the latter in this section.

### 4.1. Antimicrobial Peptides

Antimicrobial peptides (AMPs), or host defense peptides as they are colloquially known, are short peptidic fragments (fragments are typically <50 residues, full defensins are 2–6 kDa) that are employed by hosts to eliminate a broad spectrum of pathogens, including both Gram-positive and Gram-negative bacteria, fungi, and parasites [64]. AMPs are typically cationic, with charges ranging from +2 to +10, due to the high abundance of lysine and arginine residues. These highly cationic peptides interact with the negatively charged lipopolysaccharides (LPS) and lipoteichoic acid (LTA) found in bacterial cell membranes [65]. AMPs have a higher affinity for LPS and LTA than the Ca^2+^ and Mg^2+^ ions, thus, displacing them from the membrane. The displacement of these ions, along with the larger size of the peptides, helps to disrupt and destabilize the structure of the membrane. This results in the formation of pores in the bacterial membranes, subsequently causing depolarization and lysis of the cells.

**Figure 7:**
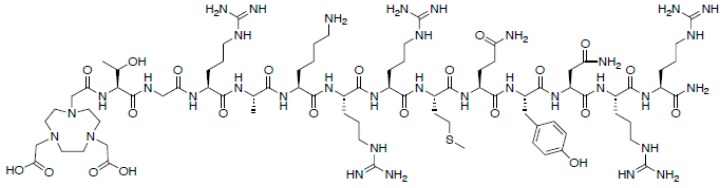
Chemical structure of NOTA-UBI_29-41._

Ubiquicidin (UBI) is the 59-residue parent AMP of UBI_29-41_, a 13-residue antimicrobial peptide fragment (Figure 7) [66,67]. This fragment of UBI binds in an electrostatic fashion with the anionic microbial cell membrane. UBI_29-41_ is perhaps the most widely studied AMP for bacterial imaging, having been directly labeled with technetium-99m, or through the use of an appended chelating ligand HYNIC. Preclinical studies of both ^99m^Tc-labeled UBI_29-41_ and [^99m^Tc]Tc-HYNIC UBI_29-41_ have shown accumulation in infected tissues with T/NT ratios of 2–3, and no accumulation in tissues with inflammation [68]. Furthermore, ^68^Ga derivatives have also been synthesized, utilizing the chelator NOTA [69,70,71]. Similar to the ^99m^Tc-labeled UBI, these ^68^Ga-labeled UBI derivatives also showed increased accumulation in bacteria (T/NT 4.0 at 1 h), without any significant increase in uptake for sterile inflammation. The success of these chelated AMPs suggests that chelated radionuclides are best suited for externally-targeted tracers.

β-defensins are a class of AMPs native to mammals, and like most AMPs, they show broad spectrum activity against Gram-positive bacteria, Gram-negative bacteria, and some fungi [64]. In a pilot study, Human β-defensin 3 (HBD-3) was labeled with ^99m^Tc, and evaluated against *S. aureus* in a rat model. The pilot study showed a modest T/NT of 2.5, while further studies have shown a T/NT of up to 5.7. Furthermore, ^99m^Tc-labeled HBD-3 was able to discriminate *S. aureus* infection from sterile inflammation [72,73,74] This again supports that chelated probes may be especially well suited for extracellular targets.

[^68^Ga]Ga-DOTA-TBIA101 is an analogue of depsidomycin [75], a naturally occurring cyclic AMP, which was investigated against *M. tuberculosis*, *E. coli*, and *S. aureus*. In a rabbit model, [^68^Ga]Ga-DOTA-TBIA101 showed weak T/NT ratios of 1.2–1.6 for *E. coli*, 1.2–1.3 for *S. aureus*, and 2.0–2.8 in *M. tuberculosis*, and the tracer also showed accumulation at sites of sterile inflammation [76,77]. It is interesting that the native cyclic-AMP showed activity against drug-resistant *M. tuberculosis*, whereas, the synthetically derived straight chain tracer was nonspecific. It is possible that by synthetically modifying the AMP to remove the macrocyclic core, the molecule is no longer as effective or specific for its target. Therefore, to ensure continued efficacy, efforts should be made to make minimal changes to the core of a molecule.

There are some limitations of AMPs, and peptide-based drugs in general, which should be noted. One challenge is the destruction of the molecule by peptidases, which cleave peptide bonds, while another is the recognition of the peptide by the immune system before the drug/probe can reach its target. One way to mitigate these issues could be macrocyclization of the peptide; however, this could also change its shape and function, much like removing the macrocycle from depsidomycin could have changed its function. Because of these issues with peptide-based drugs, many groups have devoted their research to making peptidomimetics, or peptide-like molecules, which includes: β- or γ-peptides [78,79], peptoids [80,81], spiroligomers [82,83], stapled peptides [84,85], and N-amino peptides [86,87] among many others. As there is much work on overcoming the issues associated with peptide-based drugs/therapeutics, it is important to remember new peptide-based therapeutics or diagnostics must possess an inherent ability to circumvent the immune system’s native defenses against foreign peptides or peptide fragments.

### 4.2. Antibodies

Multiple attempts to label bacterially targeted monoclonal antibodies (mAbs) have been tried, going back to the late 1980s [88] and early 1990s [89,90]. The initial results showed good T/NT ratios (~5–12); however, the clearance of the antibodies was quite slow, resulting in high background uptake, and therefore, significant T/NT ratios could only be obtained three days post-injection. More recent work has focused on the use of a human mAb (1D9), which binds the immunodominant *S. aureus* antigen A (IsaA) [91], and the use of a lipoteichoic acid (LTA) targeted anti-LTA mAb [92]. Both of these antibodies had similar issues with clearance, requiring imaging three days post-injection, and 1D9 also showed nonspecific uptake in *E. coli* and sterile inflammation, the latter believed to be the result of binding to Fc receptors on macrophages [93,94]. Another drawback to radiolabeled antibodies is that the patients are typically subjected to high levels of radiation [95]. The increased use of immunoPET, specifically fluorine-18 and zirconium-89 labeled antibodies, has been an important development over the last several years. New antibody/minibody radiotracers that target the immune cells themselves may have promise for detecting sites of infection; however, these modalities would not image the bacteria themselves [96,97,98,99,100]. One avenue that could be explored in the future is the use of minibodies or diabodies for bacterial epitopes as opposed to mAb [96,100,101,102]. The decrease in size could improve clearance times and reduce radiation burden, while having a smaller portion of the binding domain should limit off-target interactions.

## 5. Conclusions

Future endeavors should focus on the rational design of new infection imaging radiotracers. Previous efforts were hindered, in part, due to the use of chelating ligands for attachment of radionuclides, which is a suboptimal approach for intracellular targets, including antibiotic targets or those targets with complex ligand-binding mechanisms. In contrast, chelated radionuclides may be useful for extracellularly targeted radiotracers, which given the new chemistries available should be revisited in the future and with miniaturized, engineered peptides and antibodies. With greater insight and rational design, new radiotracers can be developed that allows not just the sensitive and specific diagnosis of bacterial infection, but also more depth of inquiry into the infection. For example, new probes may be able to assess the cell wall (e.g., gram positive vs. gram negative), and the particular species or resistance phenotypes (e.g., methicillin-resistant *S. aureus* vs. methicillin-sensitive *S. aureus*). Finally, all new tracers should be rigorously tested in animal models, with robust sterile inflammation controls, and ideally with and without antimicrobial therapy to show the ability to monitor the bacteria in vivo over time.

## Figures and Tables

**Table 1:** Common radionuclides used in nuclear medicine [19,20].

Isotope	T_1/2_	Attachment	Production	Decay Type	Decay Energy	Ideal Setting
^11^C	20.4 min	Covalent	Cyclotron	β+	1.0 MeV	Research/Clinical Imaging
^18^F	110 min	Covalent	Cyclotron	β+	0.6 MeV	Clinical Imaging
^68^Ga	67.6 min	Chelation	Generator	β+, γ	1.9 MeV, 1.1 MeV	Clinical Imaging *
^76^Br	16.2 h	Covalent	Cyclotron	β+	0.8–3.9 MeV	Clinical Imaging
^89^Zr	78.4 h	Chelation	Cyclotron	β+	0.9 MeV	Clinical Imaging
^90^Y	64.1 h	Chelation	Separation	β−, γ	2.3 MeV, 2.2 MeV	Therapy
^99m^Tc	6.0 h	Chelation	Generator	γ	141 keV	Clinical Imaging *
^111^In	2.8 d	Chelation	Cyclotron	γ, EC	245 keV	Clinical Imaging
^123^I	13.2 h	Covalent	Cyclotron	γ, EC	159 keV	Clinical Imaging
^124^I	4.2 d	Covalent	Cyclotron	β+	1.5–2.1 MeV	Clinical Imaging
^125^I	59.4 d	Covalent	Cyclotron	γ, EC	35 keV	Preclinical Imaging
^131^I	8.0 d	Covalent	Cyclotron	β−, γ	0.6 MeV, 364 keV	Imaging/Therapy
^177^Lu	6.7 d	Chelation	Cyclotron	β−, γ	0.5 MeV, 208 keV	Therapy

* Indicates 1–2 patients per day, per generator.

## Abbreviations

MDPI	Multidisciplinary Digital Publishing Institute
DOAJ	Directory of open access journals
PCR	Polymerase chain reaction
MRI	Magnetic resonance imaging
US	Ultrasound
CT	Computed tomography
PET	Positron emission tomography
SPECT	Single-photon emission computed tomography
DOTATATE	DOTA-octreotide
FDG	Fluorodeoxyglucose
PBP	Penicillin-binding protein
T/NT	Target-to-nontarget ratio
MIC	Minimum inhibitory concentration
PABA	*Para*-aminobenzoic acid
SMX	Sulfamethoxazole
TMP	Trimethoprim
DHFR	Dihydrofolate reductase
AMP	Antimicrobial Peptide
LPS	Lipopolysaccharide
LTA	Lipoteichoic Acid
UBI	Ubiquicidin
t_½_	Half-life
